# Molecular attributes and apoptosis-inducing activities of a putative serine protease isolated from Tiger Milk mushroom (*Lignosus rhinocerus*) sclerotium against breast cancer cells *in vitro*

**DOI:** 10.7717/peerj.4940

**Published:** 2018-06-05

**Authors:** Hui Yeng Y. Yap, Nget Hong Tan, Szu Ting Ng, Chon Seng Tan, Shin Yee Fung

**Affiliations:** 1 Department of Oral Biology, Faculty of Dentistry, MAHSA University, Kuala Lumpur, Malaysia; 2 Medicinal Mushroom Research Group, Department of Molecular Medicine, Faculty of Medicine, University of Malaya, Kuala Lumpur, Wilayah Persekutuan, Malaysia; 3 Ligno Biotech Sdn Bhd, Balakong Jaya, Selangor, Malaysia; 4 Center for Natural Products Research and Drug Discovery, University of Malaya, Kuala Lumpur, Malaysia

**Keywords:** F5, Tiger milk mushroom, Serine protease, *Lignosus rhinocerus*, Anticancer, Apoptosis, GME4347_g

## Abstract

**Background:**

The highly valued medicinal tiger milk mushroom (also known as *Lignosus rhinocerus*) has the ability to cure numerous ailments. Its anticancer activities are well explored, and recently a partially purified cytotoxic protein fraction termed F5 from the mushroom’s sclerotial cold water extract consisting mainly of fungal serine proteases was found to exhibit potent selective cytotoxicity against a human breast adenocarcinoma cell line (MCF7) with IC_50_ value of 3.00 μg/ml. However, characterization of its cell death-inducing activity has yet to be established.

**Methods:**

The mechanism involved in the cytotoxic activities of F5 against MCF7 cells was elucidated by flow cytometry-based apoptosis detection, caspases activity measurement, and expression profiling of apoptosis markers by western blotting. Molecular attributes of F5 were further mined from *L. rhinocerus*’s published genome and transcriptome for future exploration.

**Results and Discussion:**

Apoptosis induction in MCF7 cells by F5 may involve a cross-talk between the extrinsic and intrinsic apoptotic pathways with upregulation of caspase-8 and -9 activities and a marked decrease of Bcl-2. On the other hand, the levels of pro-apoptotic Bax, BID, and cleaved BID were increased accompanied by observable actin cleavage. At gene level, F5 composed of three predicted non-synonymous single nucleotide polymorphisms (T > C) and an alternative 5′ splice site.

**Conclusions:**

Findings from this study provide an advanced framework for further investigations on cancer therapeutics development from *L. rhinocerus*.

## Introduction

The World Health Organization reported that breast cancer is the most prevalent form of cancer in women both in the developed and developing world (http://www.who.int/cancer/detection/breastcancer/en/) where an estimated of 1.6 million new cases are diagnosed worldwide each year. In Malaysia, approximately 5,000 women, mostly aged between 30 and 60 years of age, are diagnosed with breast cancer annually (Cancer Research Malaysia, 2017). New cancer treatment methods and specific anticancer drugs including drug development and approval are crucial to improve treatment outcomes of this particular type of cancer.

Earlier findings from our laboratory demonstrated the selective cytotoxic effect of the sclerotial cold water extract of *Lignosus rhinocerus* TM02 against the MCF7 cell line (Yap et al., 2013), a pleural effusion derived breast (mammary gland) adenocarcinoma, thus suggesting the potential for *L. rhinocerus* TM02 to be developed as a new anticancer drug. *L. rhinocerus*, more commonly known as the tiger milk mushroom, is one of the most valuable medicinal mushrooms used by the local communities in Southeast Asia (e.g., the Semai, Temuan, and Jakun native communities in Malaysia) (Chang & Lee, 2004) to cure numerous ailments including gastric ulcers, wounds, chronic hepatitis, fever, whooping cough, asthma, cancer, and food poisoning (Jones, Hyde & Sabaratnam, 2007; Wong & Cheung, 2009). This white-rot fungus (Class: Basidiomycetes; Family: Polyporaceae) consists of a centrally stipitate pilei that arises from a submerged sclerotium which is the part with medicinal value. *L. rhinocerus* was shown to have anticancer properties that have been well explored. Lai and colleagues (2008) reported the growth inhibitory activity of a polysaccharide-protein complex from *P. rhinocerus* (synonym to *L. rhinocerus*) sclerotium against a panel of leukemic cell lines mediated by G_1_-phase cell cycle arrest. Lee et al. (2012) reported the antiproliferative effect of a sclerotial cold water extract (termed LR-CW) from *L. rhinocerotis* (synonym to *L. rhinocerus*) TM02 against MCF7 and A549 cell lines, but not in the corresponding human non-tumorigenic cell lines. Further separation of LR-CW shows that the antiproliferative activity was due to either the proteins or protein-carbohydrate complexes in the medium to high-molecular-weight fraction (Lee et al., 2012). A cold aqueous extract preparation from the sclerotium of *L. rhinocerotis* KUM61075 (LR-CA) was also shown to exhibit cytotoxicity against various human cancer cell lines, and the cytotoxic component(s) was speculated to be thermo-labile, water-soluble protein/peptide(s) (Lau et al., 2013).

We have also partially purified a cytotoxic protein fraction (termed F5) from the sclerotial cold water extract of *L. rhinocerus* TM02 that exhibited potent selective cytotoxicity against MCF7 cells with IC_50_ value of 3.00 μg/ml, as compared to its IC_50_ value on 184B5 cells at 7.60 μg/ml. The cytotoxic substance was identified to be serine protease by LC-MS/MS analysis and its proteolytic and cytotoxic activities were inhibited by phenylmethylsulfonyl fluoride, a specific serine protease inhibitor (Yap et al., 2015c). In this study, we further investigated the mechanism involved in the cytotoxic activities of this protein against MCF7 cells and based on the recently available of *L. rhinocerus* genome and transcriptome (Yap et al., 2014, 2015a), we also included more details of the F5 fraction and its link at the gene level through data mining to gain more information for future molecular exploration.

## Materials and Methods

### Mushroom material and DNA fingerprinting

The sclerotial powder of *L. rhinocerus* TM02 cultivar strain (termed TM02) was a gift from Ligno Biotech Sdn Bhd (Balakong Jaya, Selangor, Malaysia,) where consistency and quality of the production batch were verified by microbial assays and chemical profiling via HPLC-MS/MS (Batch no.: PL/1107/020). The fungus was authenticated by its nuclear ribosomal internal transcribed spacer region of ribosomal RNA according to Tan et al. (2010).

### Isolation of the cytotoxic protein fraction, F5

F5 isolation was performed according to our previous study (Yap et al., 2015c) where, in brief, cold water extraction was carried out in a mass to volume ratio of 1:20 (g/ml) at 4 °C for 24 h. The extraction mixture was then filtered and freeze dried prior to Sephadex® G-50 fractionation. Proteins from the medium-molecular-weight pooled fraction were then precipitated using 100% saturation ammonium sulfate in a mass-to-volume ratio of 1:30 (g/ml) at 4 °C for an hour and recovered with Vivaspin® 15R Centrifugal Concentrator (Sartorius Stedim Biotech, Göttingen, Germany) with 2 kDa molecular weight cut-off by centrifugation at 6,000*×g*, 4 °C followed by chromatographic fractionation by RESOURCE™ Q (1 ml) anion exchange column (GE Healthcare, Uppsala, Sweden), pre-equilibrated with start buffer (0.02 M Tris–HCl, pH 8.0). Linear NaCl gradient elution was carried out (0–100% 0.5 M NaCl in the starting buffer) at a flow rate of 1 ml/min, for 45 min. F5 was then collected and analyzed for their protease activity.

### Protease assay

Using casein as the substrate, 20 μl of F5 was mixed with 140 μl of 1% (w/v) casein in phosphate buffer (pH 7.2) prior to incubation for 15 min at 37 °C. Five percent trichloroacetic acid in 600 μl was added to the mixture prior to high-speed centrifugation for 5 min. Absorbance of the supernatant was read at 280 nm against water that acted as the blank.

### Phylogeny analysis

The protein sequence of GME4347_g was aligned to serine proteases from other related basidiomycete fungi with CLUSTAL W. Poorly aligned regions were removed by the GBlocks Server (Castresana, 2000) and PROTTEST was used to select the best fit empirical substitution model of protein evolution based on the Bayesian information criterion (Abascal, Zardoya & Posada, 2005). MEGA software version 7.0.26 was used to construct the Maximum-likelihood tree (Kumar, Stecher & Tamura, 2016).

### Cell lines and cell culture

Breast adenocarcinoma (MCF7) was purchased from ATCC® (Manassas, VA, USA). The cells were cultured in Roswell Park Memorial Institute (RPMI)-1640 (Lonza, Manassas, Basel, Switzerland) and supplemented with 10% foetal bovine serum at 37 °C in a 5% CO_2_ humidified incubator. Sub-culturing and/or media changing was performed every two to three days depending on cell confluency.

### Caspase activity measurement

Activities of caspase-8 and -9 were measured using Caspase-Glo® 8 Assay Systems and Caspase-Glo® 9 Assay Systems (Promega, Madison, WI, USA) according to manufacturer’s protocol. Luminescent signal which was proportional to caspase activity was measured an hour after the addition of Caspase-Glo® Reagent to the post-treated cells in 1:1 ratio.

### FITC Annexin V flow cytometric analysis

Apoptotic cells were quantified using BD Pharmingen™ FITC Annexin V Apoptosis Detection Kit I (BD Biosciences, Franklin Lakes, NJ, USA) according to manufacturer’s protocol. Fluorescent signal was detected with BD FACSCanto™ II cell analyzer to monitor FITC Annexin V binding and propidium iodide (PI) uptake in cells. A total of 15,000 events per sample were recorded. Data collected were analyzed with Flowing Software 2.5.1 at http://www.flowingsoftware.com/index.php?page=33 (released 4 November 2013).

### Apoptotic markers detection by western blot analysis

Post-treated cells were lysed in RIPA lysis buffer (150 mM NaCl, 1.0% Triton X-100, 0.5% sodium deoxycholate, 0.1% SDS, 50 mM Tris–HCl (pH 8.0), and two EDTA-free proteinase inhibitor cocktail tablets in a final volume of 100 ml buffer) for an hour at 4 °C prior to centrifugation to remove insoluble materials. Supernatant was then collected and the protein concentration was quantified with 2-D Quant Kit. Equal amounts of proteins (50 μg/lane) were separated by 15% SDS-PAGE and the resulting gel was transferred to a polyvinylidene difluoride (PVDF) membrane using iBlot® Transfer Stack, PVDF, mini (Novex®; Life Technologies™, Carlsbad, CA, USA) via the iBlot® Gel Transfer Device according to manufacturer’s protocol. Membrane was blocked for an hour at room temperature (RT) with 5% (w/v) non-fat dry milk in Tris buffered saline containing 0.1% (v/v) Tween-20 (TBS-T) and incubated overnight with corresponding primary antibodies (rabbit anti-human BID, Bcl-2, Bax, and β-actin) diluted to 1:1,000 in TBS-T containing 5% BSA at 4 °C. All primary antibodies were purchased from Cell Signaling Technology® (Danvers, MA, USA) and subsequently labeled with goat anti-rabbit IgG (H&L), HRP-linked antibody (Cell Signaling Technology®, Danvers, MA, USA) diluted to 1:4,000 in TBS-T containing 5% non-fat dry milk for an hour at RT. β-actin was used as a loading control. Antibody-bound protein bands were visualized by Pierce® ECL Western Blotting Substrate (Thermo Scientific, Waltham, MA, USA) according to manufacturer’s instructions and image acquisition was carried out using BioSpectrum® Imaging System (UVP, Upland, CA, USA). Densitometric analysis to determine the fold differences in protein expression was performed with myImageAnalysis^TM^ Software version 1.1 (Thermo Scientific, Waltham, MA, USA). For repeated hybridization, membrane was stripped in stripping buffer (0.4 M glycine, 2% (v/v) TBS-T, 0.2% SDS, pH 2.2) at RT for 10 min upon image acquisition.

### Statistical analysis

Results are expressed as mean ± standard deviation (SD) of three independent experiments which were performed in triplicates, unless otherwise stated. For multiple comparisons of the mean values, SPSS Statistics 17.0 (IBM, Armonk, NY, USA) with one-way ANOVA followed by LSD’s post hoc test was performed. A *p* value of less than 0.05 was considered as statistically significant.

## Results

### Molecular attributes of GME4347_g that encodes for F5

F5 contains mainly putative subtilisin-like serine proteases encoded by GME4347_g (97.29%) and GME8711_g (2.66%) as well as a small composition of lectin (0.02%) and a predicted protein (0.02%) encoded by GME272_g and GME4952_g, respectively (Yap et al., 2014, 2015c). The serine protease encoded by GME4347_g carries the conserved domains of peptidase inhibitor I9 and peptidase S8 (or subtilase) families with an Asp/His/Ser catalytic triad (Arnorsdottir, Kristjansson & Ficner, 2005; Betzel et al., 1993). The active catalytic triad sites span from 136th to 416th amino acid in GME4347_g which makes it a 281 residues sequence starting with “WGLQRISQDP,” and with a molecular weight of 28.83 kDa. The protease nature of F5 was confirmed by casein-protease assay, with trypsin as control (Yap et al., 2015c).

The full coding sequence of GME4347_g was previously reported (Yap et al., 2015c) and submitted to DDBJ/EMBL/GenBank under the Whole Genome Shotgun project version AXZM01000000 (Yap et al., 2014). This whole serine protease-encoded gene spans the 18th scaffold of the *L. rhinocerus* genome from 126543 to 128558 base pairs (bp) with 13 coding regions (435 amino acids) and estimated to have a molecular mass of 45.27 kDa (based on its protein sequence). GME4347_g is considered highly expressed and/or regulated in *L. rhinocerus* with RPKM value of 2693.41, as reported in our previous study under experiment SRX648275 (Yap et al., 2015a). Three non-synonymous single nucleotide polymorphisms (SNPs) from thymine (T) to cytosine (C) were also detected at the 126772th, 127822th, and 128424th bp of the 18th scaffold (Fig. S1) in between different samples of the same cultivar with SOAPsnp (Li et al., 2009) and an alternative 5′ splice site in the 12th coding region of GME4347_g at 128457th bp instead of the regular 5′-end at 128453th bp which could result in two different transcripts were also listed. In accordance to the generated paired-end data (Yap et al., 2015a) and further connecting the different transcriptionally active regions, this study further reports six possible extended gene models of GME4347_g as shown in Table 1.

**Table 1:** *De novo* refinement of GME4347_g structure using *L. rhinocerus* genome as reference.

Gene	5′ or 3′ end	Scaffold	Strand	Original region	Extended region
GME4347_g	5’	18	+	126543–128558	125358–125429 125465–125804 125874–126046 126088–126331 126390–126543 128558–128919

### Phylogeny analysis of GME4347_g

**Figure 1:**
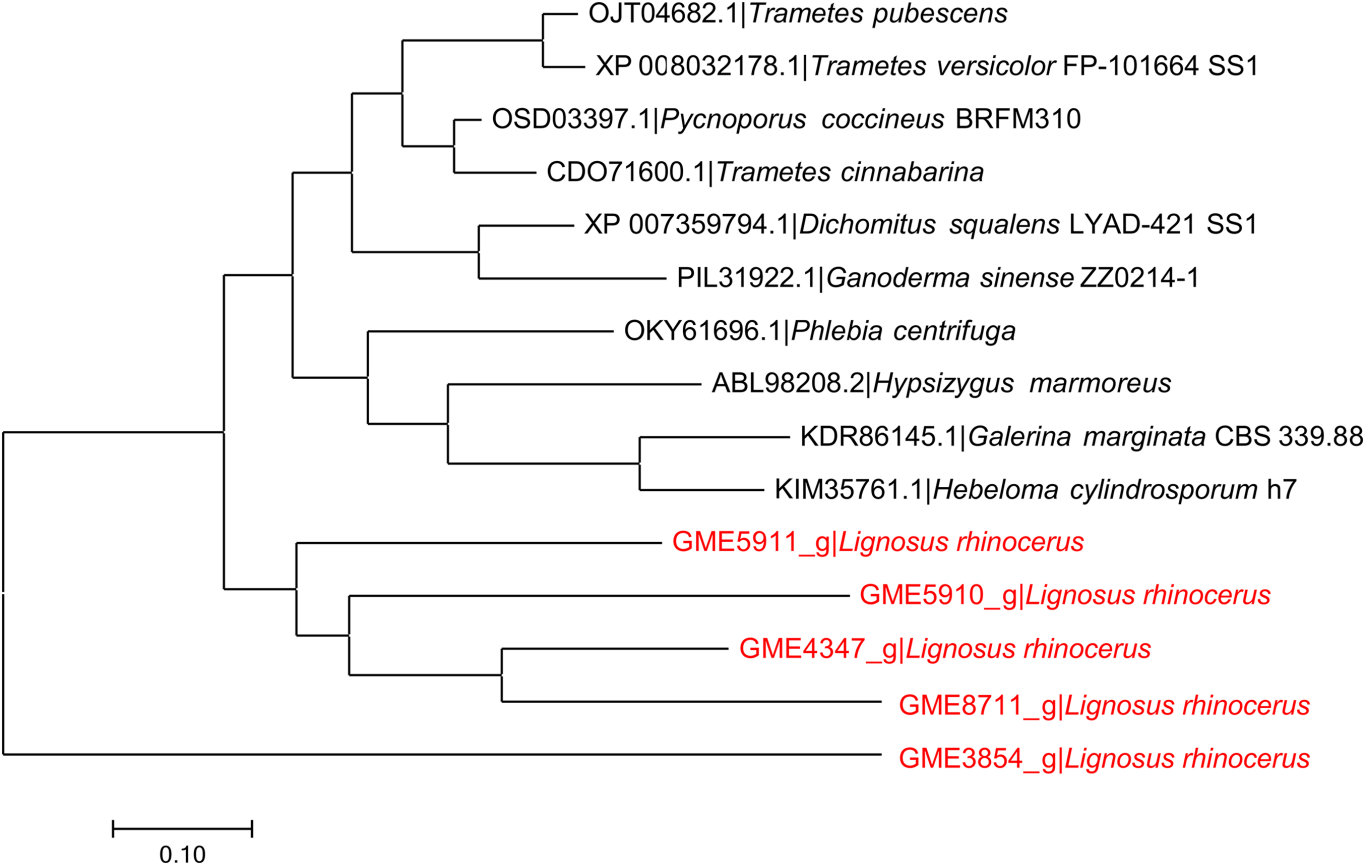
Phylogenetic relationship between putative serine proteases from *L. rhinocerus* and related proteins. JTT model based on the Bayesian information criterion (BIC) was used as the amino acid substitution model to plot the Maximum-likelihood tree with the highest log likelihood of −5424.56.

The phylogeny analysis of GME4347_g to other putative serine proteases from *L. rhinocerus* and related basidiomycete fungi (Fig. 1) showed that the five selected serine proteases annotated in *L. rhinocerus* genome are clustered together and taxonomically distant from the rest of the basidiomycetes by forming two major clades, especially for GME3854_g that also encodes for a subtilisin-like protease that carries the peptidase S8 family domain but with only 48.77% identity and 57.10% similarity to GME4347_g, and therefore is of great distance from its sub-group. The serine proteases from *L. rhinocerus* are distinctively grouped from the others, which include cuticle-degrading proteases from *Trametes pubescens* and *Phlebia centrifuga* that play a key role in cuticle penetration for fungal entomopathogenicity (Charnley & St. Leger, 1991); serine proteases *Pycnoporus coccineus*, *Dichomitus squalens*, *T. versicolor*, and *Hypsizygus marmoreus*; as well as several hypothetical proteins from *T. cinnabarina*, *Ganoderma sinense*, *Galerina marginata*, and *Hebeloma cylindrosporum*. GME4347_g was also found to show a close genetic linkage to GME8711_g with 69.41% identity and 73.78% similarity.

### Anticancer activity of F5

**Figure 2:**
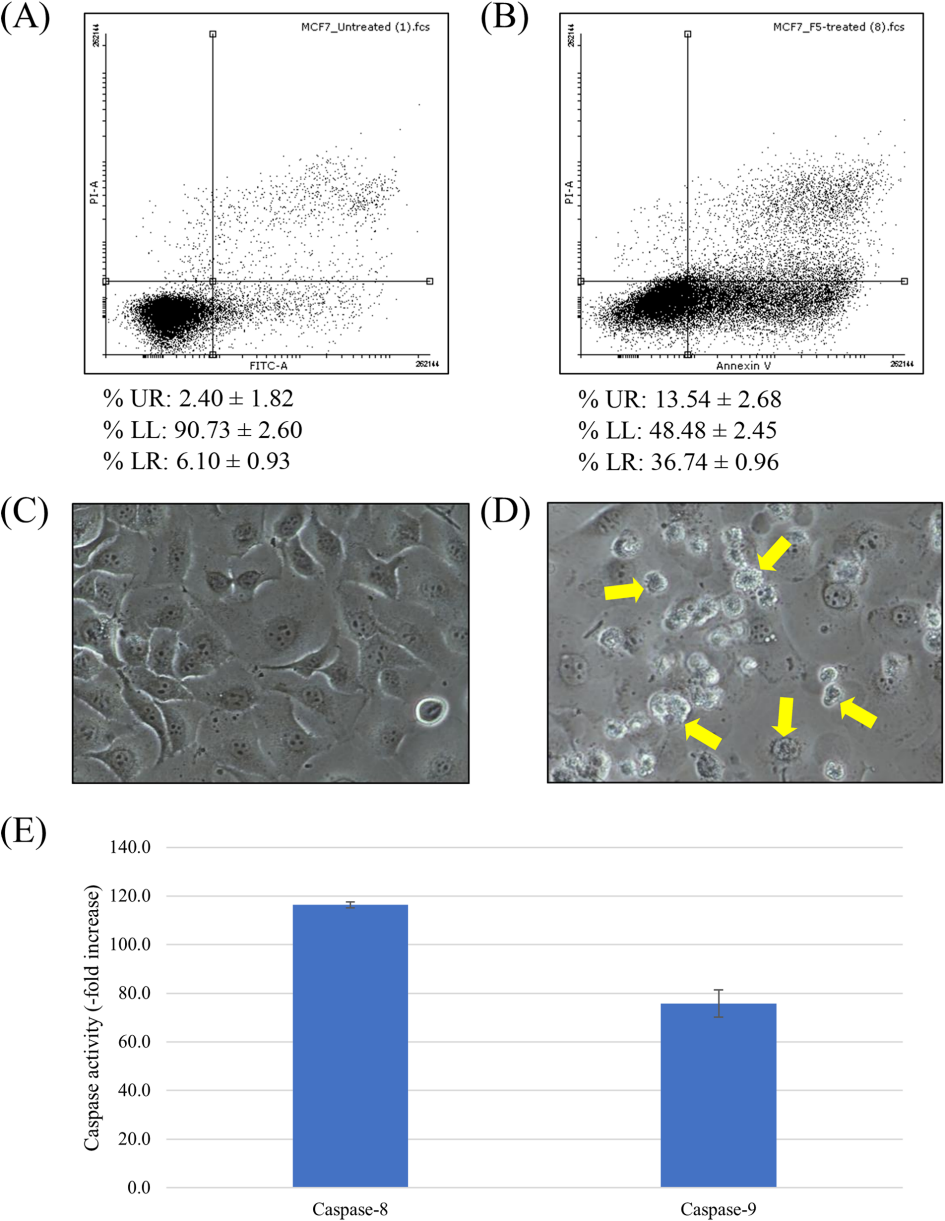
Apoptosis induction in MCF7 cells treated with F5. (A–B) Scatterplot of MCF7 cells incubated with F5 for 72 h at IC50 (3 μg/ml) (B), comparing to the untreated cells as negative control (A). Data (mean ± SD, *n* = 3) showing the percentage of cell population at UR, LL, and LR quadrants are also listed. (C–D) Cell morphology alterations of MCF7 cells upon F5 treatment at IC50 for 72 h. Apoptotic bodies were visibly distinguished in the F5-treated cells (D). Arrows showing the biochemical events of apoptosis that lead to characteristic cell changes (morphology) and death. No significant change on cell morphology was detected in cells without F5 treatment (C). Photographs of cells at 200× magnification in the same exposure. (E) Upregulation of caspases activities in MCF7 cells upon F5 treatment at 3 μg/ml (mean ± SD, *n* = 3).

In order to determine whether the cytotoxic activity of F5 is related to apoptosis induction, flow cytometric analysis using FITC Annexin V-PI staining was performed (Figs. 2A and 2B). Unlabeled cell population in the lower left (LL) quadrant represents viable cells while FITC Annexin V-labeled cells in the lower right (LR) quadrant are the apoptotic population. Upper right (UR) quadrant represents FITC Annexin V-PI-dual labeled cells, indicative of membrane permeability and late apoptosis. Our results showed that the treated MCF7 cell population skewed towards the LR quadrant, indicating apoptosis. F5 treatment leads to a significant increase in the population of FITC Annexin V-labeled early apoptotic MCF7 cells, from 6.10 ± 0.93 to 36.74 ± 0.96% (*p* < 0.05).

To further confirm that cell death is occurring via apoptosis, cellular morphology of F5-treated MCF7 cells was examined by using an inverted microscope (Figs. 2C and 2D). The F5-treated cells (Fig. 2D) possessed higher number of apoptotic bodies with notable cell morphology alterations compared to the untreated control (Fig. 2C), where a lot more of shrunken and rounded cells were detected in the former. No significant change on cell morphology was detected in cells without F5 treatment (Fig. 2C).

**Figure 3:**
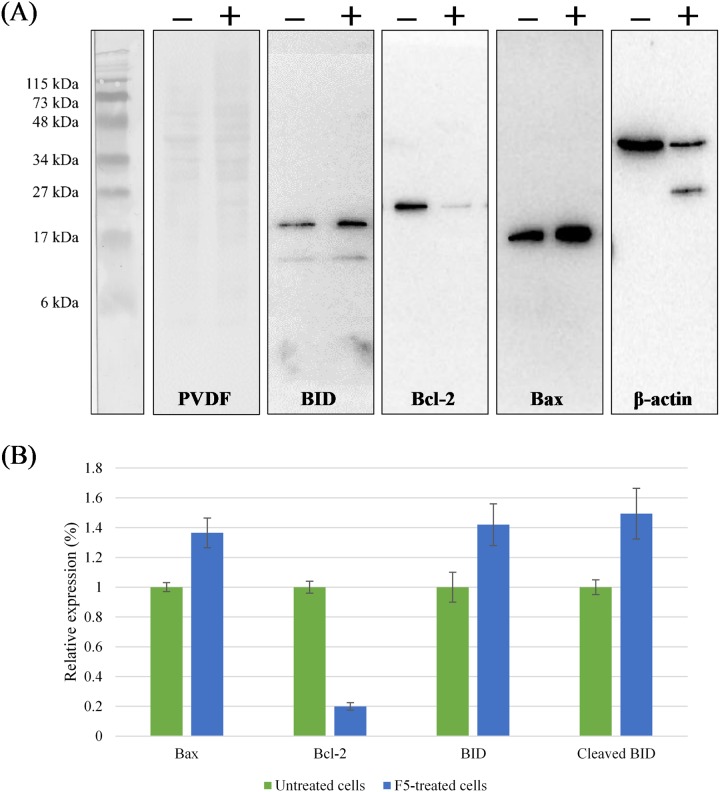
Regulation of the Bcl-2 family proteins during F5-induced apoptosis. (A) MCF7 cells were treated with F5 for 72 h at IC50, comparing to the control (Lane − vs. Lane +). The expression of BID, Bcl-2, Bax, and β-actin in the cell lysates were shown. Molecular weight of the standards (kDa) are indicated on the most left of the first panel from the left. Coomassie blue staining of PVDF membrane (50 μg protein lysates/lane) is shown in the second panel from the left. (B) Relative expression of Bcl-2 family proteins after normalization to the control (untreated cells) which was set at 1. Data expressed as mean ± SD (*n* = 2) where *p* < 0.05 in untreated cells (negative control) vs. F5-treated MCF7 cells.

The key effector role of caspase family in apoptosis is well established (Mooney et al., 2002; Nicholson & Thornberry, 1997). Activities of caspase-8 and -9 were up-regulated to a peaking 116.4- and 75.8-fold over basal levels in MCF7 cells treated with F5 at IC_50_ for 72 h, respectively (Fig. 2E) and as caspase activity was tightly regulated by a variety of factors including the Bcl-2 family proteins, we further elucidated the underlying mechanism of F5-induced apoptosis. Treated cells were examined for the expression of several Bcl-2 family proteins through western blotting (Fig. 3). F5 treatment leads to caspases-mediated actin cleavage during late apoptosis as shown in the far-right panel of Fig. 3A, demonstrating a reduction in 42 kDa β-actin to approximately 41 and 30 kDa fragments. Actin cleavage plays a positive role in the morphological changes of apoptosis downstream of the caspases activation (Fig. 2B) (Mashima, Naito & Tsuruo, 1999). F5-induced apoptosis is further accompanied by a marked decrease of Bcl-2, while the levels of Bax, BID, and cleaved BID were increased.

## Discussion

From our previous study, a cytotoxic fraction F5 was purified from the sclerotial cold water extract of *L. rhinocerus* TM02 by a combination of gel filtration chromatography, ammonium sulphate precipitation, and anion exchange chromatography (Yap et al., 2015c). F5 mainly composed of a putative subtilisin-like serine protease encoded by GME4347_g where it accounts for 97.29% of the total proteins detected in F5. GME4347_g was found to contains three SNPs with an alternative 5′ splice site in its 12th coding region (Yap et al., 2015a). However, these (SNP) variations have been thought to possibly be due to mutations (lack of stability) or genotyping error(s), which remains to be further elucidated. Furthermore, more work is needed to verify the alternative splicing event and its implication in loss- and gain-of-bioactivity of *L. rhinocerus*. Gene refinement is important to rule out the defect(s) found in vast majority of gene candidates. For instances, some genes may be corresponding to non-productive isoforms, or intrinsically broken like pseudogenes or gene fragments as well as those that are located in unfinished sequencing areas (Gotoh, Morita & Nelson, 2014). Therefore, the six possible extended gene models of GME4347_g reported in this study may serve as a platform for downstream analyses (e.g., functional implications, 3D-structure prediction of proteins, evolutionary inference of the genes and species (Gotoh, Morita & Nelson, 2014)) upon the validation of the reliability of each predicted gene structure.

The genome of *L. rhinocerus* contains several genes that code for serine proteases including GME5911_g, GME5910_g, GME4347_g, GME8711_g, and GME3854_g (Yap et al., 2014) (Fig. 1). Amino acid sequences of these genes were found to be distinctively grouped from other related basidiomycete fungi, thus reflecting dissimilarities between them, and such placement also suggests a relatively high level of divergence among these fungal serine proteases. This could also be due to the differences in their innate functions and/or substrate specificity, although further investigations will be required to ascertain this hypothesis. The close genetic linkage between GME4347_g and GME8711_g is not surprising since GME8711_g constitutes about 5.2% of the 31 kDa band in F5 (Yap et al., 2015c). Both proteins encoded by GME4347_g (10.5%) and GME8711_g (0.6%) account for 11.1% of the total proteome of *L. rhinocerus* sclerotium (Yap et al., 2015b) and thus also emerged as the most abundant serine proteases that present in the mushroom.

Most characterization of fungal serine proteases focuses on their catalytic activity, but there are very few literatures on their bioactivities. For instance, a novel alkaline serine protease, cordysobin isolated from the medicinal mushroom, *Cordyceps soboliferaa* manifested significant inhibitory activity toward HIV-1 reverse transcriptase (Wang et al., 2012) while Park et al. (2009) isolated a serine protease-like protein, designated as CMP; with antifungal and anticancer activities from the mushroom *C. militaris*. Several subtilisin-like serine proteases like saspases, phytaspases, and pspA were also found to exhibit caspase-like activity that is associated with programmed cell death for development, stress responses, and defense (Coffeen & Wolpert, 2004; Paoletti et al., 2001; Vartapetian et al., 2011). Interestingly, our earlier reported MTT analysis using F5 demonstrated that the compounds in this fraction exhibited potent selective cytotoxicity against MCF7 cells (Yap et al., 2015c) where, according to the US National Cancer Institute guidelines, a tested pure compound is considered to be an active cytotoxic agent if its IC_50_ value is less than 4 μg/ml following an incubation period of 48–72 h (Boik, 2001). The IC_50_ and IC_90_ values of F5 against MCF7 cells were reported to be 3.00 μg/ml and 4.80 μg/ml, respectively while its cytotoxicity was two-fold lower (IC_50_ = 7.60 ± 1.03 μg/ml) when tested against the non-tumorigenic 184B5 mammary gland cell line (Yap et al., 2015c). Therefore, we investigated the mechanism involved in the cytotoxic activities of this protein against MCF7 cells.

Cells undergo programmed cell death during senescence or when committed to suicide by a genetically controlled, regulated response called apoptosis. It is a normal physiological form of cell death that plays a crucial role for embryogenesis and adult tissue stem cells maintenance (Cooper & Hausman, 2004). Apoptosis kills and eliminates damaged and potentially cancerous cells. Hence, one of the fundamental processes of cancer cells is the ability to evade apoptosis, resulting in uncontrolled cell proliferation even as they accumulate mutations (Hanahan & Weinberg, 2000; van Heemst, den Reijer & Westendorp, 2007). Translocation of membrane phosphatidylserine (PS) from the inner side of the plasma membrane to the outer leaflet thereby exposing PS to the external cellular environment is one of the earlier events of apoptosis (Robers et al., 1999). In this study, the percentage of F5-treated MCF7 cells that are actively undergoing apoptosis was determined quantitatively by using FITC Annexin V, which has a high affinity for PS while PI which is membrane impermeant and has the ability to stain DNA was used as a standard viability probe to distinguish viable from non-viable cells. Based on the findings, we found that the cytotoxic activity of F5 is due to its ability to induce apoptosis and the cells undergoing apoptosis is defined by their morphological changes which involve cell shrinkage and rounding, membrane blebbing, loss of cell membrane asymmetry and attachment, chromatin condensation (pyknosis), chromosomal DNA fragmentation, as well as nuclear DNA fragmentation (karyorrhexis) leading to nucleus disintegration (Cardoso & Leonhardt, 1999). Chromatin margination and condensation occurs in the presence of preserved cellular structures and concludes with apoptotic bodies budding, thus resulting in phagocytosis of the apoptotic bodies by macrophages and neighboring cells, without any inflammatory response (Chen-Scarabelli & Scarabelli, 2004).

The ability of F5 to induce tumor cell apoptosis distinguishes it from being a toxin, but rather as a potential anticancer compound for drug development with good selectivity (Frankfurt & Krishan, 2003). Apoptosis is mediated by the integration of various signaling pathways, some acting to induce cell death while others to promote cell survival and the key effector role of caspase family in apoptosis has been well established (Mooney et al., 2002; Nicholson & Thornberry, 1997). Our results indicated that F5-induced apoptosis was mediated by caspases activation where caspase-8 and -9 are two upstream (initiator) caspases that are activated by two alternative pathways in the control of apoptosis, namely the extrinsic and intrinsic pathways, respectively (McIlwain, Berger & Mak, 2013). The former involves the mitochondria and the release of proteins from that organelle, including cytochrome *c*, apoptosis-inducing factor, and endonucleases G whereas the extrinsic pathway involves death receptors including Fas/CD95, TNFR1, DR3, DR4, and DR5 at the plasma membrane (Ashkenazi & Dixit, 1998; Hancock, 2010).

We demonstrated that activities of caspase-8 and -9 were elevated in F5-treated MCF7 cells and this was accompanied by the changes in the expression of several Bcl-2 family proteins including Bax, Bcl-2, BID, and cleaved BID. Therefore it is hypothesized that activation of caspase-7, the primary executioner caspase, was not wholly dependent on the activation of caspase-9 by cytochrome *c* release, but also via caspase-8 activation mediated by TNF family during the programmed cell death (Czabotar et al., 2014). In fact, up-regulation of caspase-8 activity was more significant compared to caspase-9. A cross-talk between the extrinsic and intrinsic pathways enacted by BID, a BH3 interacting-domain death agonist, might have existed to induce apoptosis, as supported by the results of western blot analysis. Activation of caspase-8 leads to the propagation of apoptotic signals either by direct interaction with caspase-7, or by cleavage of BID to its truncated active form, namely tBID (or cleaved BID). TBID then translocates to the mitochondria to bind directly to pro-apoptotic Bcl-2 family members, such as Bax in this case, to cause their activation. Bax promotes apoptosis by binding to and antagonizing the anti-apoptotic Bcl-2 protein where a substantial decrease of this protein was clearly observed in this study. Activation of Bax further releases cytochrome *c* from the mitochondria which in turn activates caspase-9 and initiating the caspase cascade in apoptosis (Klener et al., 2006; Kruidering & Evan, 2000; Kuida, 2000; Liang, Yan & Schor, 2001). It is known that caspase-3 is not required for apoptosis in MCF7 as the cells are caspase-3-deficient. Therefore, the mechanism appears to be: active caspase-9 activates caspase-7, which in turn activates caspase-6 that induces apoptosis in MCF7 through cleavage of nuclear lamins (Liang, Yan & Schor, 2001).

## Conclusions

A cytotoxic subtilisin-like serine protease (termed F5) isolated from *L. rhinocerus* TM02 was found to selectively inhibit the growth of MCF7 cells by inducing apoptosis. The mechanism may involve a cross-talk between the extrinsic and intrinsic apoptotic pathways. At the gene level, F5 is composed of 97.29% of serine protease that is encoded by GME4347_g carrying the conserved domains of peptidase inhibitor I9 and peptidase S8 (or subtilase) families with an Asp/His/Ser active catalytic triad. The gene is highly expressed and/or regulated in *L. rhinocerus* (RPKM value = 2693.41) with three predicted non-synonymous SNPs (T > C) and an alternative 5′ splice site. The findings from this study provide an advanced framework for further investigations on cancer therapeutics development from this mushroom. A well-designed human clinical trial is mandatory to validate the anti-cancer therapeutic benefits of this mushroom product(s) in humans. Possible future exploration should also focus on the isolation of the purified enzyme and its characterization. Alternatively, pure serine protease may be obtained by molecular cloning approach.

## Supplemental Information

10.7717/peerj.4940/supp-1 Supplemental Information 1 Raw images of blots.

10.7717/peerj.4940/supp-2 Supplemental Information 2 SNPs representation of GME4347_g in between different samples detected by SOAPsnp.

## Additional Information and Declarations

### Data Availability

The following information was supplied regarding data availability: The raw data is provided in the Supplemental File.

## References

[ref-1] Abascal F, Zardoya R, Posada D (2005). ProtTest: selection of best-fit models of protein evolution. Bioinformatics.

[ref-2] Arnorsdottir J, Kristjansson MM, Ficner R (2005). Crystal structure of a subtilisin-like serine proteinase from a psychrotrophic Vibrio species reveals structural aspects of cold adaptation. FEBS Journal.

[ref-3] Ashkenazi A, Dixit VM (1998). Death receptors: signaling and modulation. Science.

[ref-4] Betzel C, Singh TP, Visanji M, Peters K, Fittkau S, Saenger W, Wilson KS (1993). Structure of the complex of proteinase K with a substrate analogue hexapeptide inhibitor at 2.2-A resolution. Journal of Biological Chemistry.

[ref-5] Boik J (2001). Natural Compounds in Cancer Therapy.

[ref-6] Cancer Research Malaysia (2017). Facts and figures. http://www.cancerresearch.my/research/breast-cancer/.

[ref-7] Cardoso MC, Leonhardt H, Stein GS, Baserga R, Giordano A, Denhardt DT (1999). Differentiation, development, and programmed cell death. The Molecular Basis of Cell Cycle and Growth Control.

[ref-8] Castresana J (2000). Selection of conserved blocks from multiple alignments for their use in phylogenetic analysis. Molecular Biology and Evolution.

[ref-9] Chang YS, Lee SS (2004). Utilisation of macrofungi species in Malaysia. Fungal Diversity.

[ref-10] Charnley AK, St. Leger RJ, Cole GT, Hoch HC (1991). The role of cuticle-degrading enzymes in fungal pathogenesis in insects. The Fungal Spore and Disease Initiation in Plants and Animals.

[ref-11] Chen-Scarabelli C, Scarabelli TM (2004). Turning necrosis into apoptosis: the exacting task that can enhance survival. American Heart Journal.

[ref-12] Coffeen WC, Wolpert TJ (2004). Purification and characterization of serine proteases that exhibit caspase-like activity and are associated with programmed cell death in Avena sativa. Plant Cell.

[ref-13] Cooper GM, Hausman RE, Cooper GM, Hausman RE (2004). Regulation of programmed cell death. The Cell: A Molecular Approach.

[ref-14] Czabotar PE, Lessene G, Strasser A, Adams JM (2014). Control of apoptosis by the BCL-2 protein family: implications for physiology and therapy. Nature Reviews: Molecular Cell Biology.

[ref-15] Frankfurt OS, Krishan A (2003). Apoptosis-based drug screening and detection of selective toxicity to cancer cells. Anti-Cancer Drugs.

[ref-16] Gotoh O, Morita M, Nelson DR (2014). Assessment and refinement of eukaryotic gene structure prediction with gene-structure-aware multiple protein sequence alignment. BMC Bioinformatics.

[ref-17] Hanahan D, Weinberg RA (2000). The hallmarks of cancer. Cell.

[ref-18] Hancock JT, Hancock JT (2010). Life, death, and apoptosis. Cell Signalling.

[ref-19] Jones EBG, Hyde KD, Sabaratnam V (2007). Malaysian Fungal Diversity.

[ref-20] Klener P, Era L, Klener P, Necas E, Zivny J (2006). Cell death signalling pathways in the pathogenesis and therapy of haematologic malignancies: overview of apoptotic pathways. Folia biologica.

[ref-21] Kruidering M, Evan GI (2000). Caspase-8 in apoptosis: the beginning of “the end”?. IUBMB Life.

[ref-22] Kuida K (2000). Caspase-9. International Journal of Biochemistry and Cell Biology.

[ref-23] Kumar S, Stecher G, Tamura K (2016). MEGA7: molecular evolutionary genetics analysis version 7.0 for bigger datasets. Molecular Biology and Evolution.

[ref-24] Lai CKM, Wong KH, Cheung PCK (2008). Antiproliferative effects of sclerotial polysaccharides from *Polyporus rhinocerus* cooke (Aphyllophoromycetideae) on different kinds of leukemic cells. International Journal of Medicinal Mushrooms.

[ref-25] Lau BF, Abdullah N, Aminudin N, Lee HB (2013). Chemical composition and cellular toxicity of ethnobotanical-based hot and cold aqueous preparations of the tiger’s milk mushroom (*Lignosus rhinocerotis*). Journal of Ethnopharmacology.

[ref-26] Lee ML, Tan NH, Fung SY, Tan CS, Ng ST (2012). The antiproliferative activity of sclerotia of *Lignosus rhinocerus* (Tiger Milk Mushroom). Evidence-Based Complementary and Alternative Medicine.

[ref-27] Li R, Yu C, Li Y, Lam TW, Yiu SM, Kristiansen K, Wang J (2009). SOAP2: an improved ultrafast tool for short read alignment. Bioinformatics.

[ref-28] Liang Y, Yan C, Schor NF (2001). Apoptosis in the absence of caspase 3. Oncogene.

[ref-29] Mashima T, Naito M, Tsuruo T (1999). Caspase-mediated cleavage of cytoskeletal actin plays a positive role in the process of morphological apoptosis. Oncogene.

[ref-30] McIlwain DR, Berger T, Mak TW (2013). Caspase functions in cell death and disease. Cold Spring Harbor Perspectives in Biology.

[ref-31] Mooney LM, Al-Sakkaf KA, Brown BL, Dobson PR (2002). Apoptotic mechanisms in T47D and MCF-7 human breast cancer cells. British Journal of Cancer.

[ref-32] Nicholson DW, Thornberry NA (1997). Caspases: killer proteases. Trends in Biochemical Sciences.

[ref-33] Paoletti M, Castroviejo M, Begueret J, Clave C (2001). Identification and characterization of a gene encoding a subtilisin-like serine protease induced during the vegetative incompatibility reaction in *Podospora anserina*. Current Genetics.

[ref-34] Park BT, Na KH, Jung EC, Park JW, Kim HH (2009). Antifungal and anticancer activities of a protein from the mushroom *Cordyceps militaris*. Korean Journal of Physiology & Pharmacology.

[ref-35] Robers M, Rensink IJ, Hack CE, Aarden LA, Reutelingsperger CP, Glatz JF, Hermens WT (1999). A new principle for rapid immunoassay of proteins based on in situ precipitate-enhanced ellipsometry. Biophysical Journal.

[ref-36] Tan CS, Ng ST, Vikineswary S, Lo FP, Tee CS (2010). Genetic markers for identification of a Malaysian medicinal mushroom, *Lignosus rhinocerus* (Cendawan Susu Rimau). Acta Horticulturae.

[ref-37] van Heemst D, den Reijer PM, Westendorp RG (2007). Ageing or cancer: a review on the role of caretakers and gatekeepers. European Journal of Cancer.

[ref-38] Vartapetian AB, Tuzhikov AI, Chichkova NV, Taliansky M, Wolpert TJ (2011). A plant alternative to animal caspases: subtilisin-like proteases. Cell Death and Differentiation.

[ref-39] Wang S-X, Liu Y, Zhang G-Q, Zhao S, Xu F, Geng X-L, Wang H-X (2012). Cordysobin, a novel alkaline serine protease with HIV-1 reverse transcriptase inhibitory activity from the medicinal mushroom *Cordyceps sobolifera*. Journal of Bioscience and Bioengineering.

[ref-40] Wong KH, Cheung PCK, Cheung PCK (2009). Sclerotia: emerging functional food derived from mushrooms. Mushrooms as Functional Foods.

[ref-41] Yap HY, Chooi YH, Firdaus-Raih M, Fung SY, Ng ST, Tan CS, Tan NH (2014). The genome of the Tiger Milk mushroom, *Lignosus rhinocerotis*, provides insights into the genetic basis of its medicinal properties. BMC Genomics.

[ref-42] Yap H-YY, Chooi Y-H, Fung S-Y, Ng S-T, Tan C-S, Tan N-H (2015a). Transcriptome analysis revealed highly expressed genes encoding secondary metabolite pathways and small cysteine-rich proteins in the sclerotium of *Lignosus rhinocerotis*. PLOS ONE.

[ref-43] Yap HY, Fung SY, Ng ST, Tan CS, Tan NH (2015b). Genome-based Proteomic Analysis of *Lignosus rhinocerotis* (Cooke) Ryvarden Sclerotium. International Journal of Medical Sciences.

[ref-44] Yap HY, Fung SY, Ng ST, Tan CS, Tan NH (2015c). Shotgun proteomic analysis of tiger milk mushroom (*Lignosus rhinocerotis*) and the isolation of a cytotoxic fungal serine protease from its sclerotium. Journal of Ethnopharmacology.

[ref-45] Yap YH, Tan N, Fung S, Aziz AA, Tan C, Ng S (2013). Nutrient composition, antioxidant properties, and anti-proliferative activity of *Lignosus rhinocerus* Cooke sclerotium. Journal of the Science of Food and Agriculture.

