# Supplementary figures and images for: Molecular attributes and apoptosis-inducing activities of a putative serine protease isolated from Tiger Milk mushroom (Lignosus rhinocerus) sclerotium against breast cancer cells in vitro
Source: PeerJ. 2018 Jun 5;6:e4940. doi: 10.7717/peerj.4940 (PMC5993024; doi:10.7717/peerj.4940)

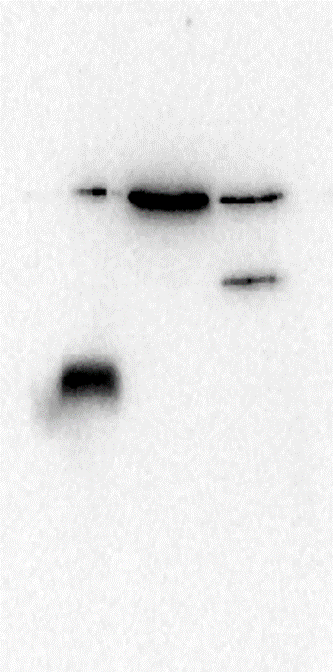

Supplement: Supplemental Information 1 [file peerj-06-4940-s001.zip › Raw data_2/Actin.png]

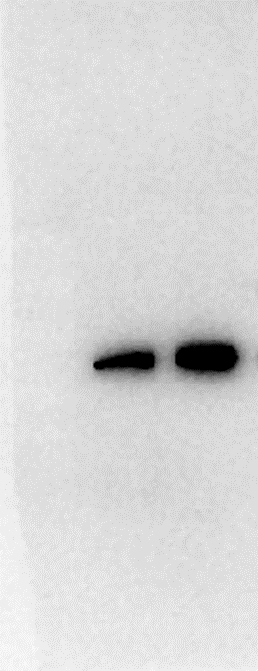

Supplement: Supplemental Information 1 [file peerj-06-4940-s001.zip › Raw data_2/Bax.png]

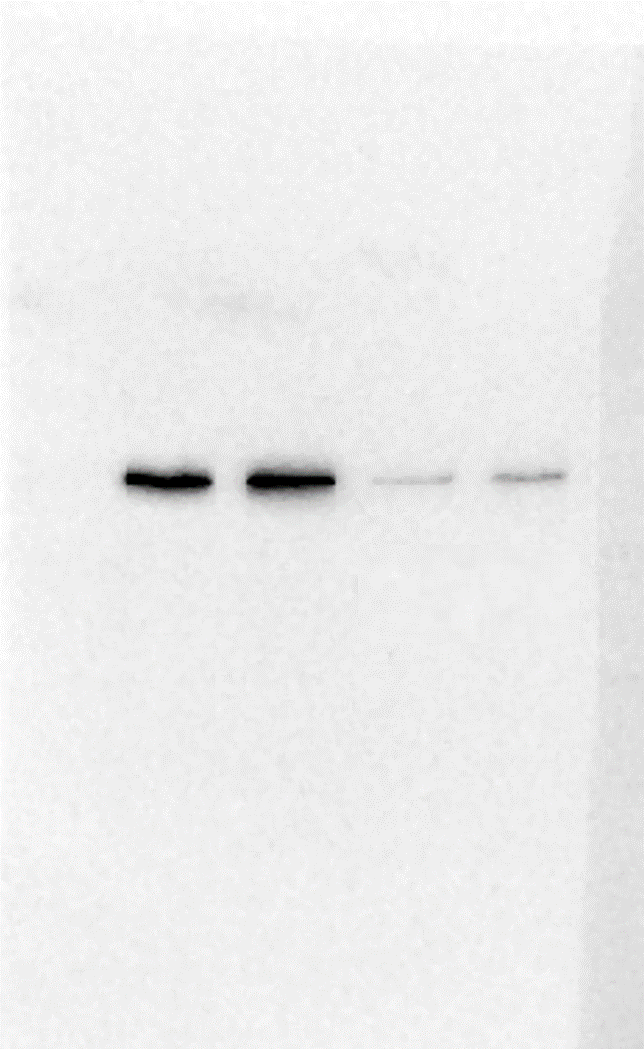

Supplement: Supplemental Information 1 [file peerj-06-4940-s001.zip › Raw data_2/Bcl-2.png]

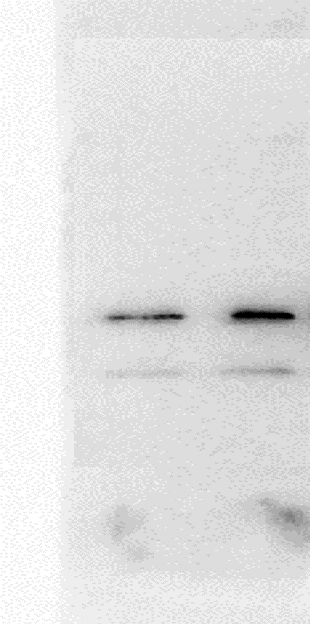

Supplement: Supplemental Information 1 [file peerj-06-4940-s001.zip › Raw data_2/BID.png]

Flowing software 2.5.1

Untreated


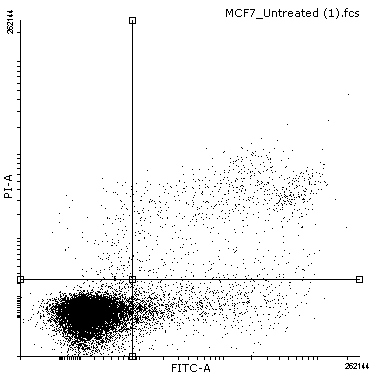

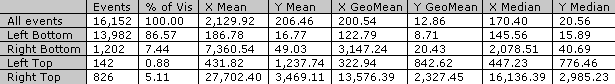


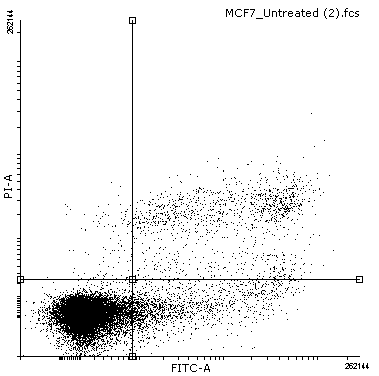


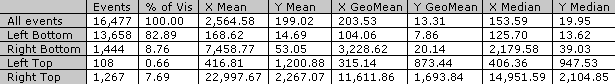


F5-treated


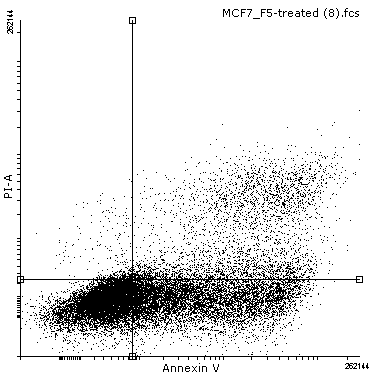


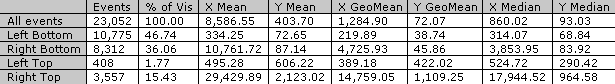


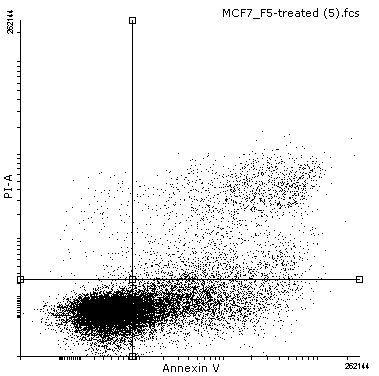


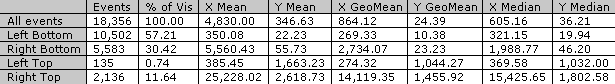

Supplement: Supplemental Information 1 [file peerj-06-4940-s001.zip › Raw data_2/FACS_exported analysis.docx]

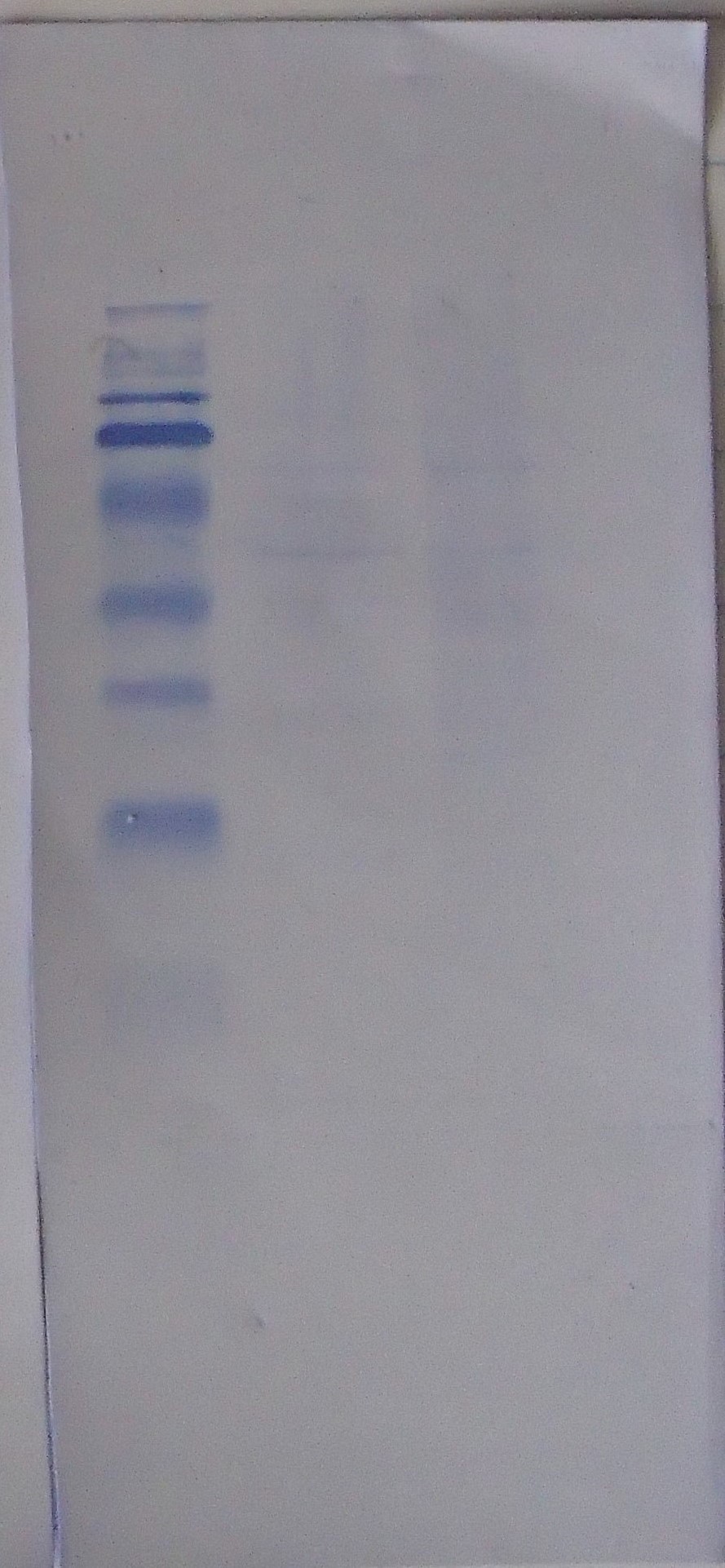

Supplement: Supplemental Information 1 [file peerj-06-4940-s001.zip › Raw data_2/Protein marker lane on PVDF membrane stained with Coomassie blue with loaded samples.JPG]

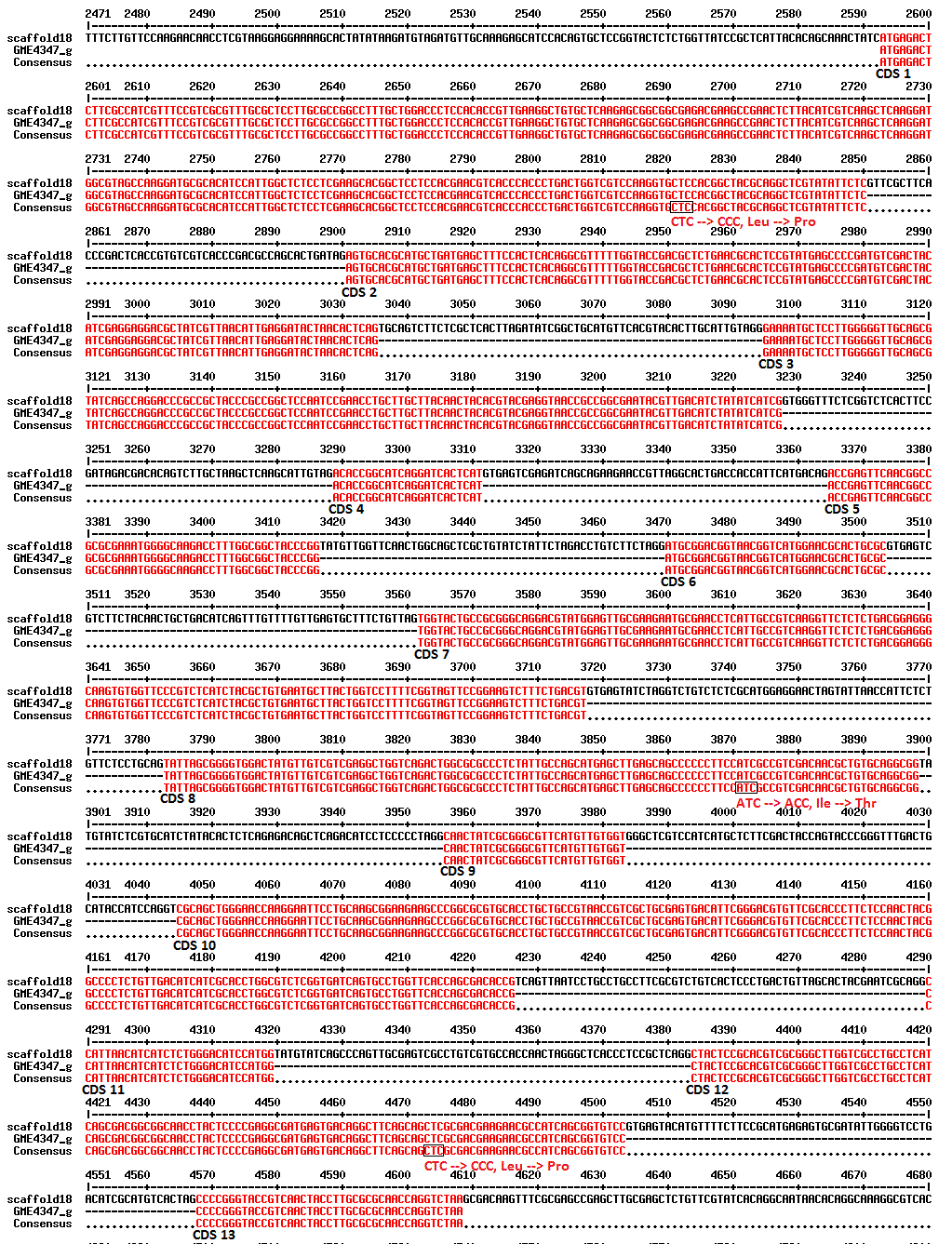

Supplement: Supplemental Information 2 [file peerj-06-4940-s002.gif]
